# Blood plasma/IgG N-glycome biosignatures associated with major depressive disorder symptom severity and the antidepressant response

**DOI:** 10.1038/s41598-017-17500-0

**Published:** 2018-01-09

**Authors:** Dong Ik Park, Jerko Štambuk, Genadij Razdorov, Maja Pučić-Baković, Daniel Martins-de-Souza, Gordan Lauc, Christoph W. Turck

**Affiliations:** 1 Max Planck Institute of Psychiatry, Department of Translational Research in Psychiatry, 80804 Munich, Germany; 2 Genos Glycoscience Research Laboratory, Zagreb, Croatia; 3 Institute of Biology, Department of Biochemistry and Tissue Biology, University of Campinas (UNICAMP), Campinas, Brazil; 4 Department of Biochemistry and Molecular Biology, Faculty of Pharmacy and Biochemistry, University of Zagreb, Zagreb, Croatia

## Abstract

While N-linked glycosylation has been extensively studied in the context of inflammatory and metabolic disorders, its relationship with major depressive disorder (MDD) and antidepressant treatment response has not been investigated. In our exploratory study, we analysed N-glycan profiles in blood plasma samples collected from MDD patients (*n* = 18) and found gender-dependent correlations with severity of depressive symptoms prior to initiating antidepressant treatment. In addition, several N-glycosylation traits showed gender-dependent associations with clinical antidepressant response. Follow up proteomics analysis in peripheral blood mononuclear cells (PBMCs) collected from MDD patients (*n* = 20) identified baseline and post-antidepressant treatment pathway differences between responder and non-responder patients. Reactome data analysis further delineated potential biological reaction differences between responder and non-responder patients. Our preliminary results suggest that specific glycosylation traits are associated with depressive symptom severity and antidepressant response and may be of use as biomarkers.

## Introduction

N-glycosylation, a post-translational modification of proteins and lipids with an oligosaccharide, modulates several biological processes including cell-cell interaction, cell adhesion, protein folding, protein localization and protein activity^1–3^. In addition, N-glycosylation plays a central role in the regulation of the immune system. The activity of one of the major effector molecules of humoral immune response, immunoglobulin G (IgG), is heavily influenced by the composition of its N-glycans. It was shown that the addition of galactose and sialic acid moieties to IgG N-glycans induces the anti-inflammatory cascade^4,5^ while the presence of core fucose regulates antibody-dependent cellular cytotoxicity (ADCC) and complement activity^6^.

Accumulating evidence suggests that a dysfunctional immune system and inflammation are associated with depressive disorders. A meta-analysis revealed that patients with major depression have fewer natural killer cells and T lymphocytes than healthy controls^7^. Increased pro-inflammatory cytokines including interleukin-1, interleukin-6 and tumour necrosis factor α have been associated with depression^8^, and have been shown to modulate the hypothalamic-pituitary-adrenal (HPA) axis as well as neurotransmitter release^9,10^.

A potential role of N-glycosylation has also been suggested for other neuropsychiatric disorders. Post-traumatic stress disorder (PTSD) patients were found to have an increased plasma agalactosylated and bigalactosylated core-alpha-1,6-fucosylated biantennary glycan ratio assessed by GlycoAge Test indicating accelerated physiological aging^11^. In schizophrenia patients glutamate transporters, excitatory amino acid transporters 1 and 2 (EAAT1 and EAAT2), were found to be less glycosylated in the prefrontal cortex^12^ and genes involved in N-glycan biosynthesis showed altered expression^13^.

Several glycosylation enzymes have been strongly implicated in psychiatric disorder pathobiology. Total plasma sialyltransferase levels in combination with cortisol were shown to reflect HPA axis function^14^. Treatment with dexamethasone, a synthetic corticosterone, was shown to stimulate sialyltransferase hepatic transcription and serum activity^15,16^. The sialyltransferase gene *ST6GALNAC1* promoter region was significantly hypomethylated in psychosis-associated twins compared to healthy twins^17^. The *ST8SIA2* gene that encodes α−2, 8-sialyltransferase 8B was shown to associate with increased risk to mental illness^18^. Mannosyltransferase gene disruption was found in a family with bipolar affective disorder history^19^. Beta-1,3-galactosyltransferase gene *B3GALT5* SNPs showed a strong association with schizoaffective and bipolar disorders^20^.

Proteomics has been widely used to identify affected proteins and pathways in major depressive disorders (MDD), which has provided insights into molecular mechanisms of MDD and its treatment. Energy metabolism and synaptic pathways have repeatedly been associated with MDD^21–24^ and based on our recent studies also with the antidepressant treatment response^25–27^.

Proteomic and N-glycomic profiling analysis results are further strengthened by interrogating manually curated and peer-reviewed reactome databases^28,29^ that include various biological events, pathways and reactions to infer affected biological pathways, thereby adding important functional information^30,31^.

In the present study, we asked the question whether blood plasma/IgG N-glycome and peripheral blood mononuclear cells (PBMCs) proteome biosignatures are associated with depressive symptom severity and antidepressant treatment response. For this purpose we analysed blood plasma total protein and IgG N-glycome profiles from MDD patients at baseline and 6 weeks after antidepressant treatment. In addition, we acquired proteomics data from MDD patients’ PBMCs.

## Results

### Plasma/IgG N-glycome profiling in MDD patients

N-glycome of total plasma proteins and IgG were analysed in 18 depressed individuals (responder, n = 10; non-responder, n = 8) at two time points, prior to admission to the clinic (T0) and 6 weeks after chronic antidepressant treatment (T6) (Supplementary Table 1). Total plasma N-glycome was analysed by HILIC-UPLC and resulted in N-glycans that were separated in 39 chromatographic peaks from which 13 derived traits were calculated. IgG N-glycome of distinct subclasses (IgG1, IgG2/3, and IgG4) was analysed by LC-ESI-MS and resulted in 20 glycoforms and 7 derived traits per subclass (Supplementary Table 2).

**Figure 1:**
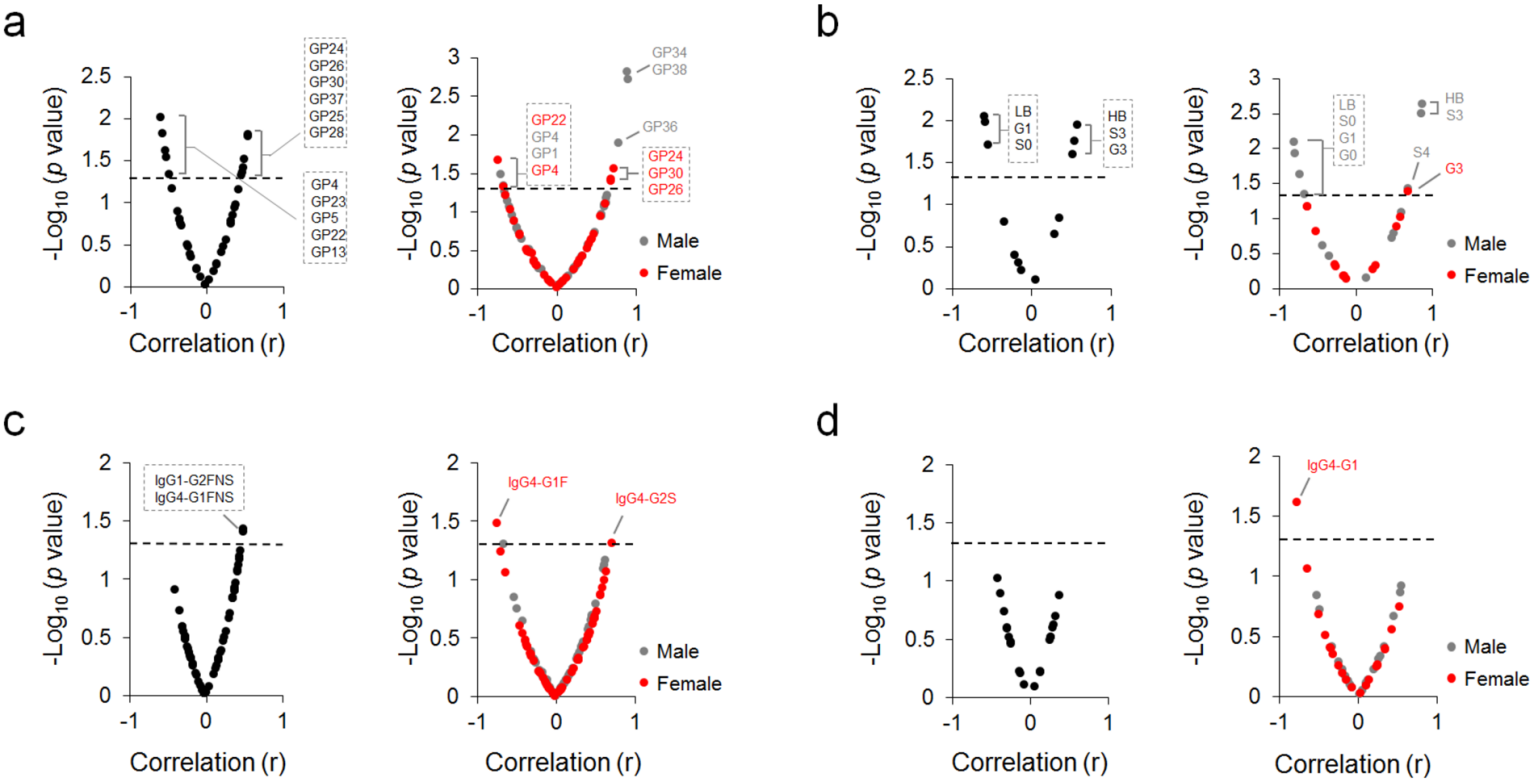
Correlation of baseline N-glycosylation traits with severity of depression. Correlation of (**a**) glycan peaks (GPs) and (**b**) derived traits in total plasma proteins with HDRS score at T0, left panel: all cohorts (*n* = 18), right panel: gender sub-groups (male, n = 9; female, n = 9). Correlation of IgG subclasses’ (IgG1, IgG2/3 and IgG4) (**c**) glycoforms and (**d**) derived traits with HDRS score at T0, left panel: all cohorts (*n* = 17), right panel: gender sub-groups (male, *n* = 9; female, *n* = 8). One non-responder MDD patient sample was excluded from IgG N-glycomic analysis due to a measurement failure. Pearson and Spearman correlations were calculated based on normal distribution test outcome. GPs and N-glycosylation traits with−Log10(*p*-value) > 1.3 were considered significant, corresponding to *p* < 0.05.

We examined the relationship between N-glycan profiles and Hamilton Depression Rating Scale (HDRS) at T0 to find out whether baseline glycan species are associated with patients’ depressive symptom severity. In all cohorts, 11 glycan peaks were significantly correlated with T0 HDRS score (Fig. 1a, left panel). Male and female MDD patients showed differential glycan peak profiles correlated with T0 HDRS score (Fig. 1a, right panel). While 6 derived traits of total plasma proteins were correlated with T0 HDRS score in all cohorts (Fig. 1b, left panel), 7 derived traits in male and 1 trait in female patients correlated with T0 HDRS score (Fig. 1b, right panel). Specifically, highly branched N-glycan structures showed significant positive correlations with T0 HDRS score. In case of the IgG N-glycome, significant correlation with depressive symptom severity was found only for two galactosylated and sialylated glycoforms containing bisecting N-acetylglucosamine in all cohorts (Fig. 1c, left panel). Female patients had 2 IgG4 N-glycan glycoforms significantly correlated with T0 HDRS score, whereas male patients did not have significantly correlated IgG glycoforms (Fig. 1c, right panel). No significant correlations were found between IgG trait levels and T0 HDRS score in all cohorts (Fig. 1d, left panel). Only IgG4-G1 trait was correlated with T0 HDRS score in female patients (Fig. 1d, right panel).

### PBMC proteome profiling in MDD patients

**Figure 2:**
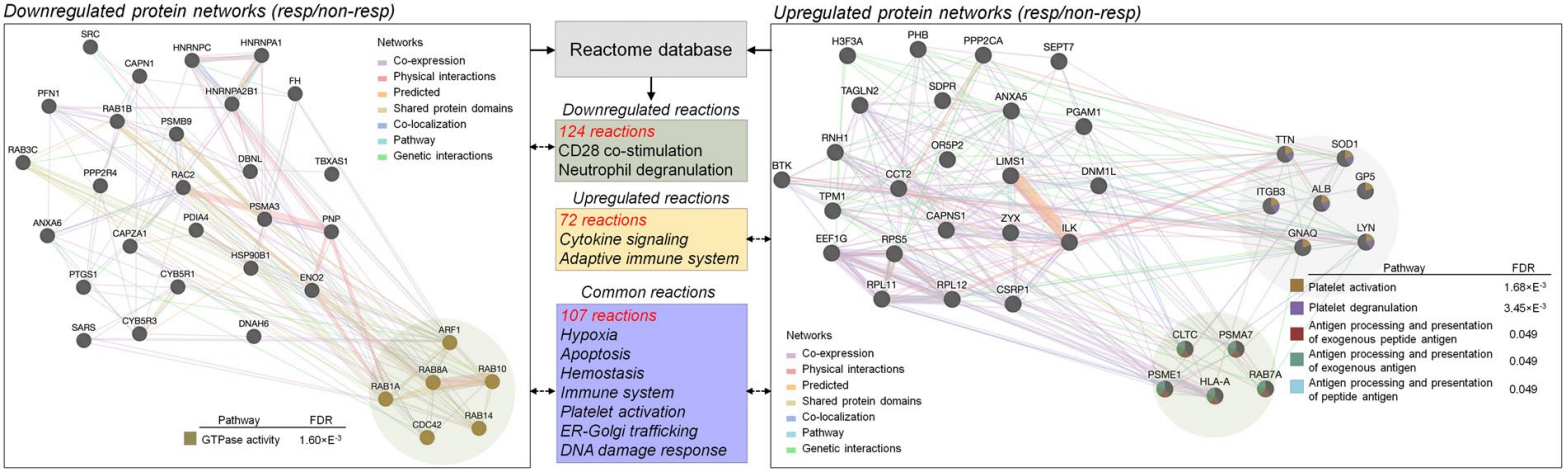
Baseline proteome and reactome differences between antidepressant responder and non-responder MDD patients prior to the admission to the clinic. Differentially expressed PBMC proteins between antidepressant responder (*n* = 12) and non-responder patients (*n* = 8) in all cohorts significantly enriched several pathways (FDR < 0.05). Proteome-associated biological reactions were analysed based on reactome database (FDR < 0.05). No gender specific analysis was performed in PBMC proteomics data.

To further explore baseline biological features which might be predictive for the antidepressant response, we analysed previously acquired^32^ PBMC proteome data from antidepressant responder (n = 12) and non-responder (n = 8) MDD patients that had undergone 6 weeks of antidepressant treatment (Fig. 2). PBMC proteomics data were acquired from pooled samples of the same cohorts. Baseline GTPase activity pathway was downregulated in the antidepressant responder group. Proteins significantly upregulated in the antidepressant responder group at baseline enriched 5 pathways related to platelet activation and antigen processing and presentation (FDR < 0.05).

Reactome pathway analysis further corroborated various process alterations related to MDD. Biological reactions including hypoxia, apoptosis, immune system, platelet activation, ER-Golgi trafficking and DNA damage response were commonly enriched when comparing antidepressant responder and non-responder groups (FDR < 0.05).

### Antidepressant response-related plasma/IgG N-glycome

**Figure 3:**
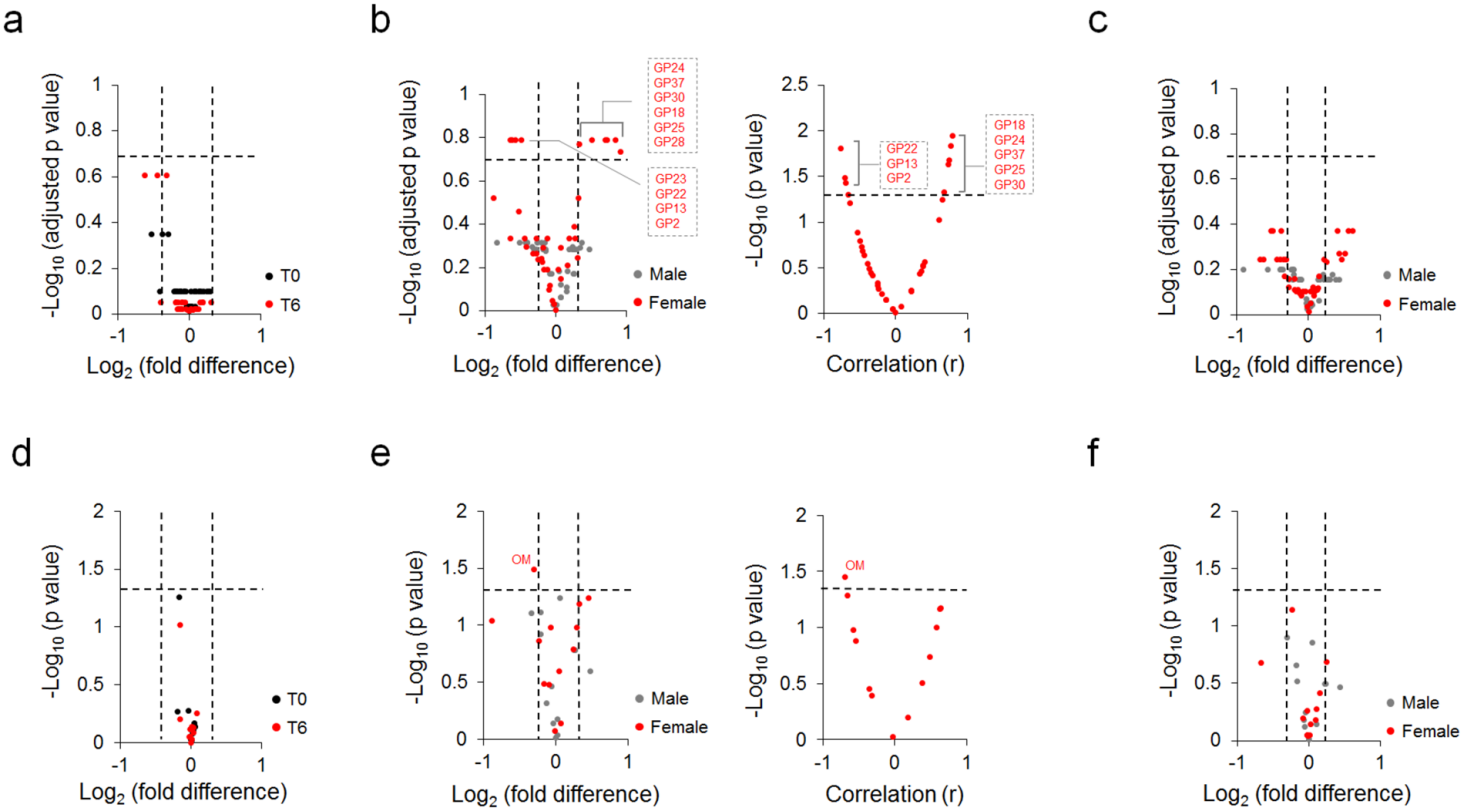
Plasma glycoform and derived trait differences between responder and non-responder patients. (**a**) Plasma glycoform differences between responder and non-responder patients both at T0 and T6, *n* = 18 in all cohorts. (**b**) Gender specific differences of plasma glycoforms (left panel: male *n* = 9, female *n* = 9) and correlation of female glycoforms with clinical antidepressant response (right panel: *n* = 9) at T0. (**c**) Gender specific glycoform differences at T6, male *n* = 9, female *n* = 9. (**d**) Plasma glycosylation trait differences between responder and non-responder patients both at T0 and T6, *n* = 18 in all cohorts. (**e**) Gender specific differences of plasma glycosylation traits (left panel: male *n* = 9, female *n* = 9) and correlation of plasma N-glycosylation trait in female patients with clinical antidepressant response (right panel: *n* = 9) at T6. (**f**) Gender specific differences of glycosylation trait at T6, male *n* = 9, female *n* = 9. GPs with log2|fold difference| > 0.3 and –log10(adjusted *p* value) > 0.7 were considered significant, corresponding to >20% fold differences and FDR < 0.2. N-glycosylation traits with log2|fold difference| > 0.3 and –log10(*p* value) > 1.3 were considered significant, corresponding to > 20% fold differences and *p* < 0.05. Pearson and Spearman correlations were calculated based on normal distribution test outcome. Correlations with−log10(*p* value) > 1.3 were considered significant, corresponding to *p* < 0.05. T0, prior to admission to the clinic; T6, after 6 weeks of antidepressant treatment.

We also evaluated the relationship between total plasma proteins’/IgG N-glycosylation and HDRS score change after 6 weeks of chronic antidepressant treatment. Correlation between T0 N-glycosylation and HDRS score change was examined to identify glycosylation traits which could predict clinical antidepressant response. Correlation between T6 N-glycosylation and HDRS score change was investigated to find glycosylation traits with potential diagnostic value for the antidepressant response. In plasma, all study cohorts showed no significant GP level differences between responder and non-responder groups both at T0 and T6 (Fig. 3a). However, 10 GP levels at T0 were significantly different between female responder and non-responder groups (FDR < 0.2) (Fig. 3b, left panel). Eight female GPs showed a significant correlation between their baseline levels and HDRS score change (Fig. 3b, right panel). None of male and female T6 GPs were correlated with HDRS score change (Fig. 3c).

While all cohorts showed no plasma protein N-glycan trait differences between responder and non-responder groups both at T0 and T6 (Fig. 3d), one derived trait at T0, oligo-mannose N-glycan levels, was different between female responder and non-responder patients (FDR < 0.2) (Fig. 3e, left panel) and correlated negatively with HDRS score change (*p* < 0.05) (Fig. 3e, right panel). None of male and female traits at T6 associated with antidepressant efficacy in MDD patients (Fig. 3f).

**Figure 4:**
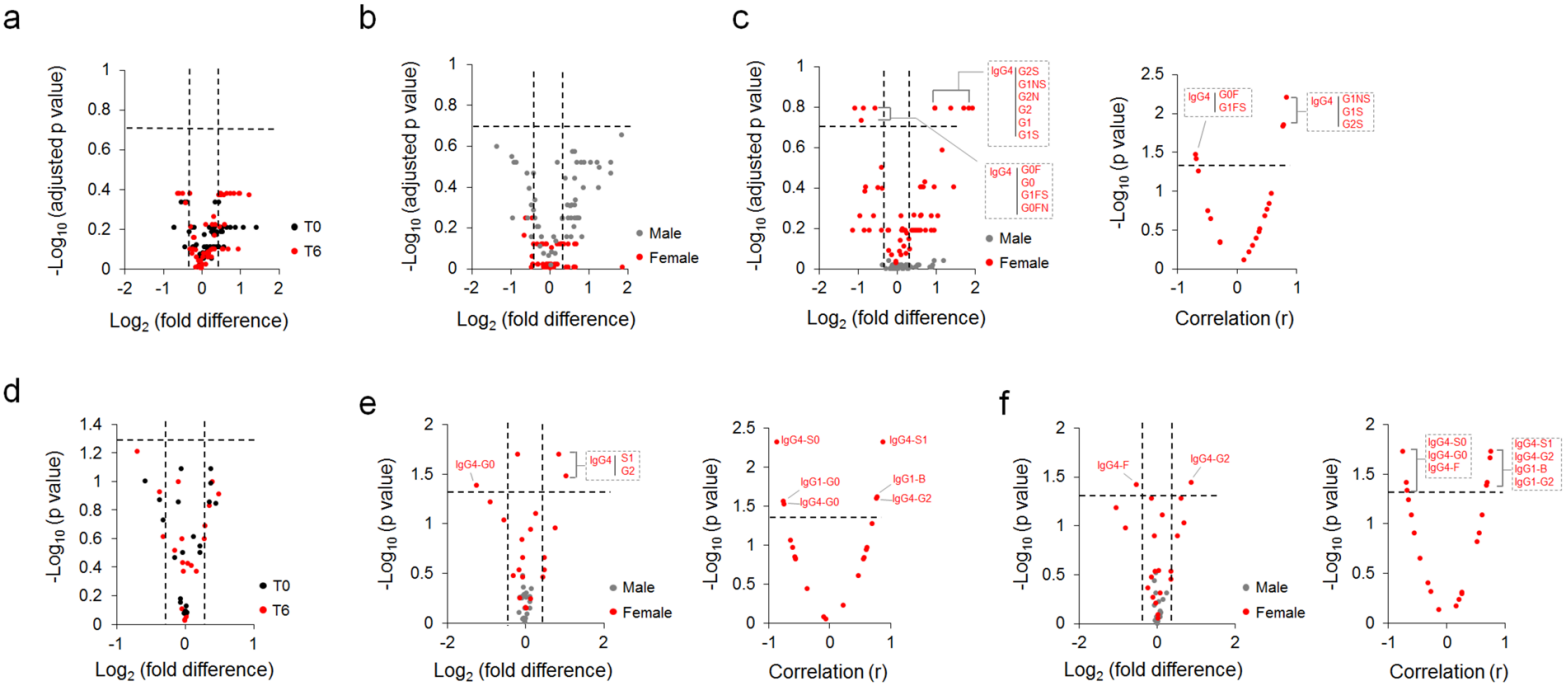
IgG glycoform and derived trait differences between responder and non-responder patients. (**a**) IgG N-glycoform differences between responder and non-responder patients both at T0 and T6, T0 *n* = 17, T6 *n* = 18 in all cohorts. One non-responder MDD patient sample at T0 was excluded from IgG N-glycomic analysis due to a measurement failure. (**b**) Gender specific differences of IgG N-glycoforms between responder and non-responder patients at T0, male *n* = 9, female *n* = 8. (**c**) Gender specific IgG N-glycoform level differences between responder and non-responder groups (left panel: male *n* = 9, female *n* = 9) and correlation of female IgG N-glycoforms with clinical antidepressant response at T6 (right panel: *n* = 9). (**d**) IgG N-glycosylation trait differences in overall cohorts between responder and non-responder patients both at T0 and T6, T0 *n* = 17, T6 *n* = 18. (**e**) Gender specific IgG glycosylation trait differences (left panel: male *n* = 9, female *n* = 8) and correlation of female IgG traits with clinical antidepressant response (right panel: *n* = 8) at T0. (**f**) Gender specific IgG glycosylation trait differences (left panel: male *n* = 9, female *n* = 9) and correlation of female IgG traits with clinical antidepressant response (right panel: *n* = 9) at T6. IgG Glycoforms with log2|fold difference| > 0.3 and−log10 (adjusted *p* value) > 0.7 were considered significant, corresponding to > 20% fold differences and FDR < 0.2. IgG N-glycosylation traits with log2|fold difference| > 0.3 and−log10 (*p* value) > 1.3 were considered significant, corresponding to >20% fold differences and *p* < 0.05. Pearson and Spearman correlations were calculated based on normal distribution test outcome. Correlations with−log10 (*p* value) > 1.3 were considered significant, corresponding to *p* < 0.05. T0, prior to admission to the clinic; T6, after 6 weeks of antidepressant treatment.

For IgG N-glycan profiles, all cohorts showed no differences between responder and non-responder patients both at T0 and T6 (Fig. 4a). Neither male nor female sub-groups showed IgG glycoform level differences between responder and non-responder patients at T0 (Fig. 4b). After chronic antidepressant treatment, 10 female IgG4 glycoforms were different between the two groups (FDR < 0.2) (Fig. 4c, left panel) and 5 female IgG4 glycoform levels were significantly correlated with HDRS score change (*p* < 0.05) (Fig. 4c, right panel). While no differences were observed for IgG N-glycosylation traits both at T0 and T6 in all cohorts (Fig. 4d), 3 female IgG4 N-glycosylation traits were significantly different between responders and non-responders at T0 (Fig. 4e, left panel) and correlated with HDRS score change (Fig. 4e, right panel). Chronic antidepressant treatment resulted in IgG4-F and G2 trait level differences (FDR < 0.2) and significant correlation (*p* < 0.05) in female patients (Fig. 4f). Male patients had no significantly different IgG N-glycan glycoforms and traits between responder and non-responder groups.

### Antidepressant response-related PBMC proteome

**Figure 5:**
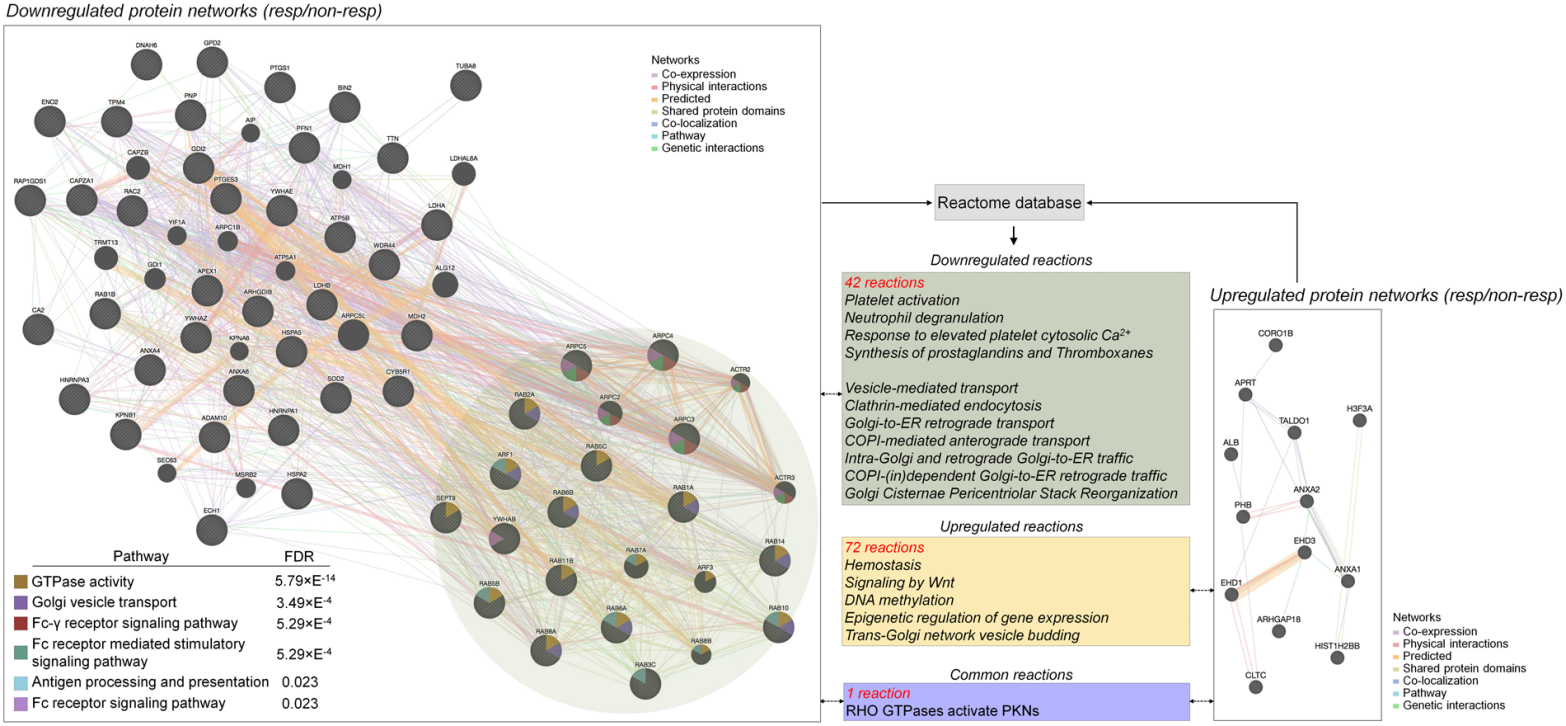
Proteome and reactome analyses between antidepressant responder and non-responder patients after 6 weeks of antidepressant treatment. Differentially expressed PBMC proteins between responder (*n* = 12) and non-responder patients (*n* = 8) in overall cohorts significantly enriched several pathways (FDR < 0.05). Proteome-associated reactions were analysed based on reactome database (FDR < 0.05). No gender specific analysis was performed in PBMC proteomics data.

We also investigated proteomic signatures related to antidepressant treatment response^32^. PBMCs obtained after 6 weeks of antidepressant treatment were compared between drug responder and non-responder MDD patients (Fig. 5). Six biological pathways including GTPase activity, Golgi vesicle transport, antigen processing and presentation, and Fc receptor-associated pathways were significantly downregulated in antidepressant responders when compared to non-responders (FDR < 0.05). Reactome database search results indicated pathological pathway downregulation including platelet activation and neutrophil degranulation. In addition, a downregulation of several ER-Golgi pathways in responder patients was found. No biological pathways were enriched by upregulated proteins in responders. Reactome analysis indicated that responder patients are characterized by 72 upregulated reactions including hemostasis, Wnt signaling, epigenetic regulation and trans-Golgi network vesicle budding.

To investigate the relationship between identified N-glycan signatures and proteome/reactome pathways, integrin β3 and NF-κB proteins which play a central role in platelet activation and inflammatory pathway, respectively, were investigated. We found that several male plasma N-glycan traits including LB, G1, S0, HB and S3 were significantly correlated with integrin β3 levels at T0 (*p* < 0.05) (Table 1 and Supplementary Figure 1a). Male plasma LB trait was also significantly correlated with NF-kB protein levels at T0. In addition, female correlations between N-glycan trait and pathway protein levels at T6 were assessed with regard to antidepressant treatment response (Table 2 and Supplementary Figure 1b). While significantly different N-glycan traits did not show a correlation, IgG1-B was significantly correlated with NF-κB protein levels, which indicates that IgG1-B might be associated with inflammatory pathway regulation (*p* < 0.05). Correlations between Integrin β3 levels and other glycan traits including IgG1-G2, IgG4-S0 and IgG4-S1 only showed a trend (*p* < 0.1).

**Table 1:** Correlation of integrin β3 and NF-κB protein levels with significantly different blood plasma/IgG N-glycan traits at T0.

*Gender*	*Traits (T0)*		*Integrin β3*	*p value*	*NF-*κB	*p value*
			*r*		*r*	
Male	plasma	LB	−0.78	0.013	−0.67	0.049
		G0	−0.63	0.069	−0.27	0.481
		G1	−0.77	0.016	−0.45	0.224
		S0	−0.73	0.026	−0.37	0.325
		HB	0.79	0.012	0.58	0.992
		S3	0.74	0.022	0.56	0.118
Female	plasma	G3	0.21	0.619	0.25	0.551
	IgG4	G1	−0.68	0.062	0.25	0.555

Cropped and full-length blots of integrin β3 and NF-κB proteins are presented in Supplementary Figure 1a.

**Table 2:** Correlation of integrin β3 and NF-κB protein levels with significantly different blood plasma/IgG N-glycan traits at T6.

*Gender*	*Traits (T6)*		*Integrin β3*		*NF-*κB	
			*r*	*p value*	*r*	*p value*
Female	IgG1	B	−0.55	0.162	0.74	0.037
		G2	−0.66	0.075	0.29	0.492
	IgG4	S0	0.67	0.069	−0.29	0.485
		S1	−0.67	0.069	0.29	0.485
		F	0.54	0.17	−0.42	0.306
		G2	−0.61	0.107	0.27	0.51

Cropped and full-length blots of integrin β3 and NF-κB proteins are presented in Supplementary Figure 1b.

We further evaluated the effect of age on N-glycan profiles (Supplementary Figure 2). While a great number of plasma/IgG N-glycosylations were correlated with age, only IgG1-G0 showed an overlap with antidepressant response-related N-glycosylation profiles.

## Discussion

**Figure 6:**
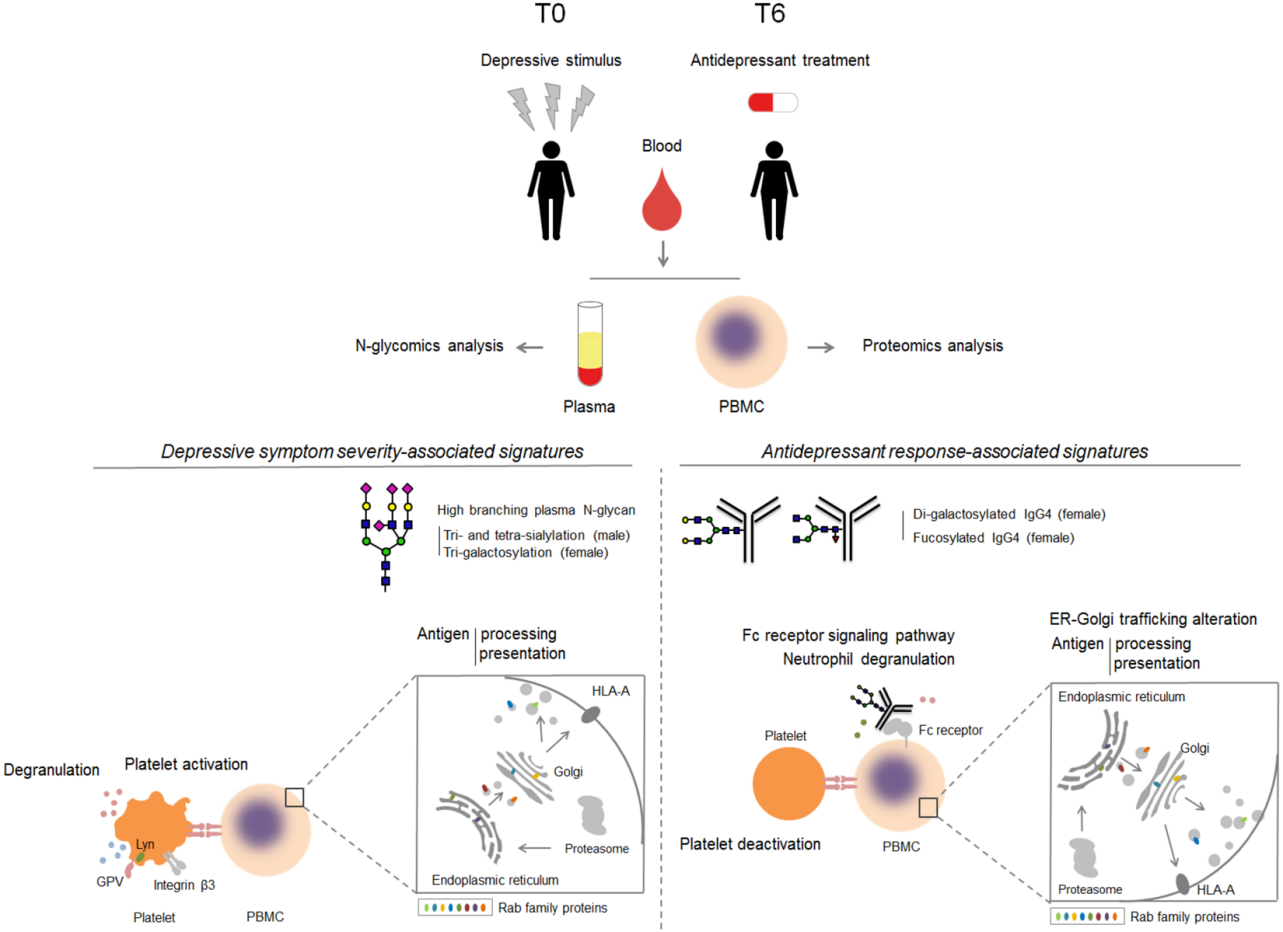
The overview illustrating plasma/IgG N-glycosylation traits and PBMC proteome/reactome pathways associated with depressive symptom severity and antidepressant treatment response.

Our preliminary findings suggest that blood plasma/IgG N-glycan and PBMC protein signatures might be associated with depressive symptom severity and antidepressant treatment response (Fig. 6). Furthermore, analysis of PBMC proteomics data previously obtained from MDD patients^32^ identified biological pathway differences between antidepressant responders and non-responders.

N-glycosylation has been implicated in MDD aetiology in previous studies. Yamagata *et al*. found that plasma glycan Sia-α2–6Gal/GalNAc was altered both in a mouse model of depression and MDD patients’ leukocytes^33^. The authors also found decreased α-N-acetylgalactosaminide α−2,6-sialytransferase 2 protein expression levels in patients’ leukocytes indicating a dysregulated sialylation process in MDD pathophysiology. Ding *et al*. reported elevated plasma galactose levels in MDD compared to healthy controls and suggested that galactose may contribute to glycan formation and is possibly linked with inflammation^34^.

We found that several ER and Golgi associated proteins, including Arf and Rab, were differentially expressed between antidepressant responder and non-responder patients. Arf and Rab have been implicated in protein glycosylation by their ability to regulate ER and Golgi vesicles carrying glycosylation related enzymes^35,36^, in our case possibly resulting in differential glycosylation traits and activity between antidepressant responder and non-responder MDD patients.

In our previous studies we found proteins that are part of the integrin and ras signalling pathways to be differentially expressed between antidepressant responder and non-responder patients^32^. Plasma proteomics analysis also revealed differential fibrinogen α levels between drug responders and non-responders^37^. These results are in line with the present data since integrin, ras and fibrinogens are crucial for platelet activation^38^. Platelet activation is a cardiovascular risk factor in depressed patients^39^. Activated platelets secrete various factors including platelet factor 4, P-selectin and several inflammatory mediators accompanying morphological changes and aggregation^40^.

Sialic acid has been shown to be a ligand for P- and E-selectins which modulate platelet activation^41^. Furthermore, Mercado *et al*. found that the serotonin-induced platelet N-acetyl-neuraminic acid to N-glycolyl-neuraminic acid switch contributes to platelet activation^42^. The here identified N-glycan signatures may thus be a representation of differential platelet activation between responder and non-responder patients.

Since serotonin and glutamate are key regulators for platelet activation^43,44^, our observation that responders showed greater baseline platelet activation than non-responders may be a reflection of higher basal capacity for response to antidepressant treatment. Supporting this observation, responders had downregulated biological reactions associated with platelet activation after chronic antidepressant treatment.

Our preliminary data also suggest that successful antidepressant treatment may downregulate immune system activation. In line with our study, antidepressants have been shown to decrease the inflammatory response^45,46^. IgG Fc sialylation has been shown to be anti-inflammatory^4,47^ and galactosylated glycans were shown to be essential for binding with galectins which have been suggested to play a crucial role in the regulation of neuroinflammation^48,49^.

Based on significant correlations between plasma N-glycan traits and integrin β3/NF-κB proteins at T0, plasma protein glycosylation activity at baseline might be important for platelet activation and inflammatory pathway regulation with regard to depressive symptom severity. Since only IgG1-B showed a significant correlation with NF- κB protein levels at T6, further studies including other pathway proteins are necessary. A direct assessment of platelet activation in patient samples is required for further validation of a functional relationship between blood plasma/IgG N-glycome and PBMC proteome and its significance for the antidepressant response.

Due to the small number of patient specimens and the fact that patients received different antidepressant medications our study suffers from low statistical power. However, since our previous studies with the same patient cohorts showed consistent molecular pathway differences between responders and non-responders^25,26^, pharmacological heterogeneity in our cohorts may not be critical. We also found little overlap between age-related and treatment response-related N-glycans/traits. This may indicate that age does not impact antidepressant response-associated N-glycans/traits in our cohorts.

PBMC samples of each group were pooled for relative protein quantitation. This may also cause low statistical power of the analyses, requiring validation in larger cohorts. Nevertheless, our proteomics data are in line with previous observations. Integrin β3 polymorphism has been suggested to be associated with differential antidepressant response^50^. Annexin A2 protein has been associated with chronic selective serotonin reuptake inhibitor (SSRI) response^51^. This indicates that our proteomics data may provide valuable information that supports earlier studies.

Lack of control for other parameters which can affect glycosylation process is another limitation of this study. Body mass index, smoking and lipid profiles including cholesterol and triglycerides have been shown to be associated with various glycosylation patterns including fucosylation, sialylation and galactosylation^52–55^. Whether they impact MDD and antidepressant response-related N-glycan profiles will be investigated in a future study.

Identified N-glycosylation and proteome signatures were not acquired from a healthy control group. However, the here identified signature patterns correlated with depressive symptom severity or antidepressant treatment response. Biosignatures from N-glycome and proteome might be used to predict and diagnose the severity of depression and antidepressant efficacy.

## Materials and Methods

Details for PBMC proteomics analysis, functional pathway enrichment analysis, reactome pathway analysis and western blot analysis are provided as a part of ‘Supplementary Information’.

### Human plasma and PBMC preparation

Human blood plasma and PBMCs were collected from 38 depressed participants of the ‘Munich Antidepressant Response Signature’ study at baseline (T0) and 6 weeks after antidepressant treatment (T6) (Supplementary Table 1). PBMCs were isolated using Histopaque reagent (Sigma-Aldrich, St Louis, MO, USA). Briefly, fresh blood was subjected to Accuspin Histopaque cartridge (Sigma-Aldrich) and centrifuged (1000 g, RT, 15 min). After collecting plasma, a layer containing PBMCs was carefully transferred to a new tube, Histopaque reagent added and centrifuged (1000 g, RT, 15 min). The PBMCs were washed three times with phosphate buffered saline (PBS) and centrifuged to pellet (800 g, RT, 15 min). The PBMC pellet was stored at −80 °C for further analysis. Diagnosis was conducted according to Diagnostic and Statistical Manual of Mental Disorders, 4^th^ Edition (DSM-IV) criteria. Depression severity was evaluated using the 21-item Hamilton Depression Rating Scale (HDRS). Patients with at least moderate depression severity as indicated by HDRS score > 14 at entry were included. Responder patients were classified based on their clinical antidepressant treatment response corresponding to a minimal 50% reduction of the HDRS score between T0 and T6. The ‘Munich Antidepressant Response Signature’ project was approved by the ethics committee of the Medical Faculty of the Ludwig Maximilians University Munich, Germany (submission number 318/00). Informed consent was obtained from all participants included in the study. All procedures were carried out in accordance with the approved guidelines.

### Plasma glycan release and labelling

Plasma samples (5 μl) were denatured by adding 20 μl 2% SDS (w/v) (Invitrogen, Carlsbad, CA, USA) and incubating at 65 °C for 10 min. After incubation, samples were left to cool down to room temperature for 30 min. Subsequently, 10 μl of 4% Igepal-CA630 (v/v) (Sigma-Aldrich) was added to the samples and incubated on a shaker for 15 min. Then 1.2 U of PNGase F (Promega, Madison, WI, USA) in 10 μl 5 × PBS was added and incubated overnight at 37 °C for N-glycan release. The released N-glycans were labelled with 2-aminobenzamide (2-AB) (Sigma-Aldrich). The labelling mixture was freshly prepared by dissolving 2-AB in DMSO (Sigma-Aldrich) and glacial acetic acid (Merck, Darmstadt, Germany) mixture (70:30, v/v) and by adding 2-picoline borane (2-PB) (Sigma-Aldrich) to a final concentration of 19.2 mg/mL for 2-AB and 44.8 mg/ml for 2-PB. Twenty five μl of labelling mixture was added to each N-glycan sample in the 96-well plate and the plate was sealed using adhesive tape. Mixing was achieved by shaking for 10 min, followed by 2 h incubation at 65 °C.

Free label and reducing agent were removed from the samples by hydrophilic interaction liquid chromatography solid-phase extraction (HILIC-SPE) using 0.2 μm GHP filter plates (Pall Corporation, Ann Arbor, MI, USA) as described previously^56^.

### HILIC-UPLC

Fluorescently labelled plasma N-glycans were separated by HILIC on a Waters Acquity ultra-performance liquid chromatography (UPLC) instrument (Milford, MA, USA) with a quaternary solvent manager, sample manager and a FLR fluorescence detector set with excitation and emission wavelengths of 250 and 428 nm, respectively. The instrument was under the control of Empower 3 software, build 3471 (Waters). Labelled plasma N-glycans were separated on a Waters BEH Glycan chromatography column, 150 × 2.1 mm i.d., 1.7 μm BEH particles, with 100 mM ammonium formate, pH 4.4, as solvent A and acetonitrile as solvent B and a 23 min linear gradient of 70–53% acetonitrile (v/v) at a flow rate of 0.56 ml/min. Samples were maintained at 10 °C before injection, and the separation temperature was 25 °C. The system was calibrated using an external standard of hydrolyzed and 2-AB labelled glucose oligomers from which the retention times for the individual glycans were converted to glucose units. Data processing was performed using an automatic processing method with a traditional integration algorithm after which each chromatogram was manually corrected to maintain the same intervals of integration for all the samples. The chromatograms were all separated in the same manner into 39 peaks and the amount of glycans in each peak was expressed as % of total integrated area. List of the most abundant N-glycan structures present in each chromatographic peak is available as Supplementary Table 3.

### Isolation of IgG

The IgG was isolated from human plasma using protein G monolithic plates (BIA Separations, Ajdovščina, Slovenia) as described previously^57^. Briefly, 50–100 µl of plasma was diluted 8× with 1 × PBS, pH 7.4, applied to the protein G plate and instantly washed with 1× PBS, pH 7.4 to remove unbound proteins. IgGs were eluted with 1 ml 0.1 M formic acid (Merck) and neutralized with 1 M ammonium bicarbonate (Merck).

### Trypsin digestion and reversed phase-solid phase extraction (RP-SPE)

IgG was digested with trypsin to obtain Fc glycopeptides as previously described^58^. To 25 μg of protein, 0.2 μg of sequencing grade trypsin (Promega) was applied, sealed with thermal foil and incubated overnight at 37 °C. After trypsin digestion, glycopeptides were purified using reversed-phase (RP) SPE to remove salts and impurities. Trypsin digests were diluted 10 × with 0.1% TFA (v/v) (Sigma-Aldrich) and loaded onto 5 mg of Chromabond C-18 ec sorbent (Macherey-Nagel, Düren, Germany). Samples were washed three times with 200 μl 0.1% TFA, eluted with 200 μl 20% ACN (v/v) and completely dried in centrifugal evaporator (Thermo Scientific, Waltham, MA, USA).

### LC-ESI-MS analysis

IgG Fc glycopeptides were analysed by liquid chromatography online mass spectrometry (LC-MS) as previously described^59^ with slight changes. Glycopeptides were separated on Waters nanoACQUITY LC system coupled to Compact Q-TOF MS (Bruker Daltonik, Bremen, Germany). Dried glycopeptides were reconstituted in 20 μl ultrapure water. Sample volume of 9 μl was loaded onto Thermo PepMat C-8 5 × 0.3 mm i.d., 5 μm particle size trap column followed by a 1 min wash with solvent A (0.1% TFA) at a flow rate of 40 μl/min. After washing, glycopeptides were separated on Halo C-18 150 mm × 0.1 mm i.d., 2.7 μm particle size analytical column based on differences in their peptide backbone applying a 3.5 min gradient from 18% to 25% solvent B (0.02% TFA in 80% ACN). Column temperature was maintained at 30 °C and flow rate was 1 μl/min. Column outlet was connected to the mass spectrometer via CE sprayer (Agilent Technologies, Santa Clara, CA, USA) to which 2 μl/min flow of sheathing liquid (50% IPA, 20% propionic acid, 30% H_2_O) was constantly applied. The mass spectrometer was operated under the following conditions: spectra between 600 m/z and 1800 m/z were recorded at a frequency of 0.5 Hz; drying nitrogen gas was set to 4 L/min and 180 °C, while nebulizing gas was set to 0.4 bar. Collision energy was 4 eV with argon as collision gas. Capillary voltage was 4500 V. NanoAcquity and Compact were operated under HyStar software version 3.2, build 44 (Bruker). Obtained spectra were converted to centroid mzXML data files using DataAnalysis software version 4.1, build 362.7 (Bruker). The 20 most abundant glycopeptides for each IgG subclass (IgG1–4) were extracted. Extraction was performed with an in-house developed Python 3 script. In short, quantitative m/z targets were calculated for 20 different glycoforms of 3 LC/MS separable IgG isotypes (IgG1, IgG2/3 and IgG4), for doubly and triply protonated species and for top 4 isotopologues, individually. Since IgG2 and IgG3 glycopeptides have the same amino acid backbone in the Caucasian population^60^, they were exported together as IgG2/3. Measured data outside of 4 m/z window around individual targets were filtered out.

m/z domain was re-calibrated using a hand-picked set of glycopeptide targets from random 20 samples, which had an expected isotopic pattern and were clear from interference. For every sample and preselected calibrant, the top 3 signals from 4 m/z window around the calibrant and most intense scan were picked as matching measured data. Calibrants were validated based on expected charge state, S/N and inlying of relative m/z errors based on interquartile range. Cubic spline was fitted to validate calibrants and matching measured data per sample using UnivariateSpline class from scipy.interpolate package. Intensities were extracted from recalibrated data as base peak intensities from 10 ppm m/z window around every quantitative m/z target per scan per sample. Integration retention time bins corresponding to chromatographically separated IgG isotypes were manually defined from overlayed extracted ion chromatograms. For every sample and quantitative target extracted intensities from corresponding integration bin were summed. Glycopeptide quantities were derived from summed areas of all corresponding charge states and isotopologues.

### Statistical Analysis

N-glycosylation data were normalized with sum of values. Derived traits, i.e. N-glycosylation patterns such as levels of galactosylation and sialylation were calculated using formulas shown in Supplementary Table 3. N-glycome differences between responder and non-responder patients were analysed with two-tailed t test followed by false discovery rate (FDR) correction. FDR was also applied for multiple correction of proteome and reactome pathway enrichment. D’Agostino’s normality test was used to check normal distribution of data. Pearson and Spearman correlations were calculated using GraphPad Prism 5 (GraphPad Software, Inc., La Jolla, CA, USA). No outliers were detected with Grubbs’ test in the analyses of each N-glycoform and derived trait.

### Data availability

The datasets generated and/or analysed during the current study are available from the corresponding author on reasonable request.

## Electronic supplementary material

Supplementary Information

Supplementary Table 1

Supplementary Table 2
