# Supplementary material for: Blood plasma/IgG N-glycome biosignatures associated with major depressive disorder symptom severity and the antidepressant response
Source: Sci Rep. 2018 Jan 9;8:179. doi: 10.1038/s41598-017-17500-0 (PMC5760622; doi:10.1038/s41598-017-17500-0)
Supplement: Supplementary file 1 — Supplementary Information [file 41598_2017_17500_MOESM1_ESM.doc]

# Supplementary Information

**Title: Blood plasma/IgG N-glycome biosignatures associated with major depressive disorder symptom severity and the antidepressant response**

Dong Ik Park^1^, Jerko Štambuk^2^, Genadij Razdorov^2^, Maja Pučić-Baković^2^, Daniel Martins-de-Souza^3^, Gordan Lauc^2,4^, Christoph W. Turck^1*^.

^1^ Max Planck Institute of Psychiatry, Department of Translational Research in Psychiatry, 80804, Munich, Germany; ^2^ Genos Glycoscience Research Laboratory, Zagreb, Croatia.; ^3^ Institute of Biology, Department of Biochemistry and Tissue Biology, University of Campinas (UNICAMP), Campinas, Brazil; ^4^ Department of Biochemistry and Molecular Biology, Faculty of Pharmacy and Biochemistry, University of Zagreb, Zagreb, Croatia.

***Corresponding author**:

Prof. Dr. Christoph W. Turck

Max Planck Institute of Psychiatry, Department of Translational Research in Psychiatry, Kraepelinstrasse 2-10, 80804 Munich, Germany

Phone: +49-89-30622317

E-mail: [turck@psych**.**mpg.de](mailto:turck@psych.mpg.de)

**Supplementary Results**


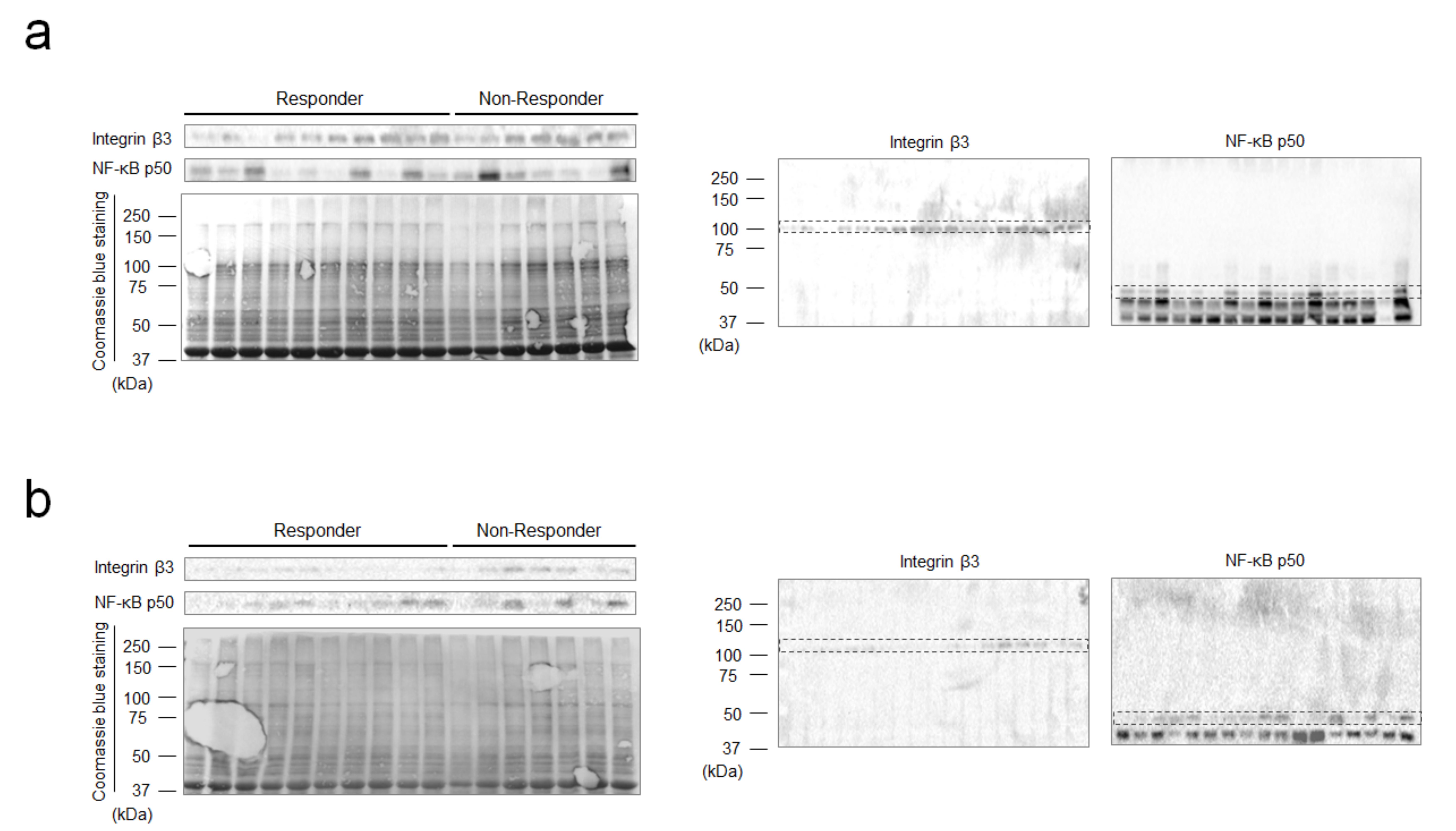


**Supplementary Figure 1.** Integrin β3 and NF-κB Western blots obtained from MDD patients’ PBMCs (responders: *n=*10; non-responders: *n=*7) at (**a**) T0 and (**b**) T6. Full-size Western blot images are presented in the right panel. Coomassie Brilliant Blue staining was used as loading control.


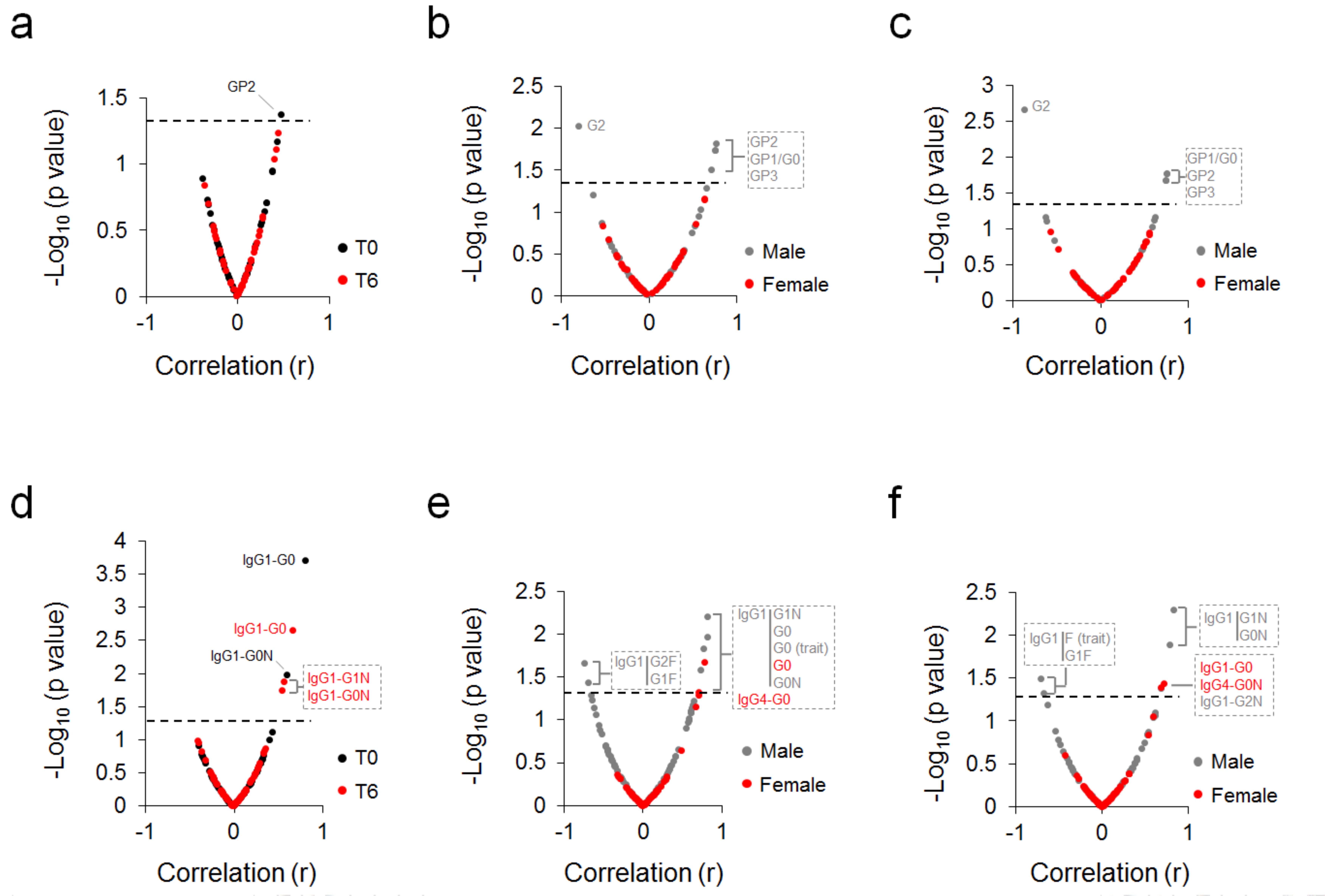


**Supplementary Figure 2.** (**a**) Correlation between age and plasma N-glycan profiles at T0 and T6 in overall cohorts, *n=*18. Gender-specific correlations between age and plasma N-glycan profiles at (**b**) T0 and (**c**) T6, male= 9; female=9. (**d**) Correlation between age and IgG N-glycan profiles in overall cohorts, *n=*17. Gender-specific correlations between age and IgG N-glycan profiles at (**e**) T0 and (**f**) T6, male=9; female=8.

**Supplementary Table 1**. Patient information. HDRS, Hamilton Depression Rating Score. T0: at clinical admission; T6: after six weeks of admission. TCA: Tricyclic antidepressant; SSRI: Selective serotonin reuptake inhibitor; SNRI: Serotonergic and noradrenergic reuptake inhibitor; NASSA: Noradernergic and selective serotonergic antidepressant; NARI: noradrenergic reuptake inhibitor; SSRE: Selective serotonin reuptake enhancer; OTH: Other antidepressants; NL: antipsychotic co-medication; PP: mood stabilizing co-medication; LI: lithium co-medication; BZD: Benzodiazepine co-medication; SLP: sleep enhancing medication. 0.00 = no. 1.00 = yes. NPE, Numbers of Previous Episodes. N/A, Not Available.

**Supplementary Table 2.** Plasma/IgG N-glycan raw data.


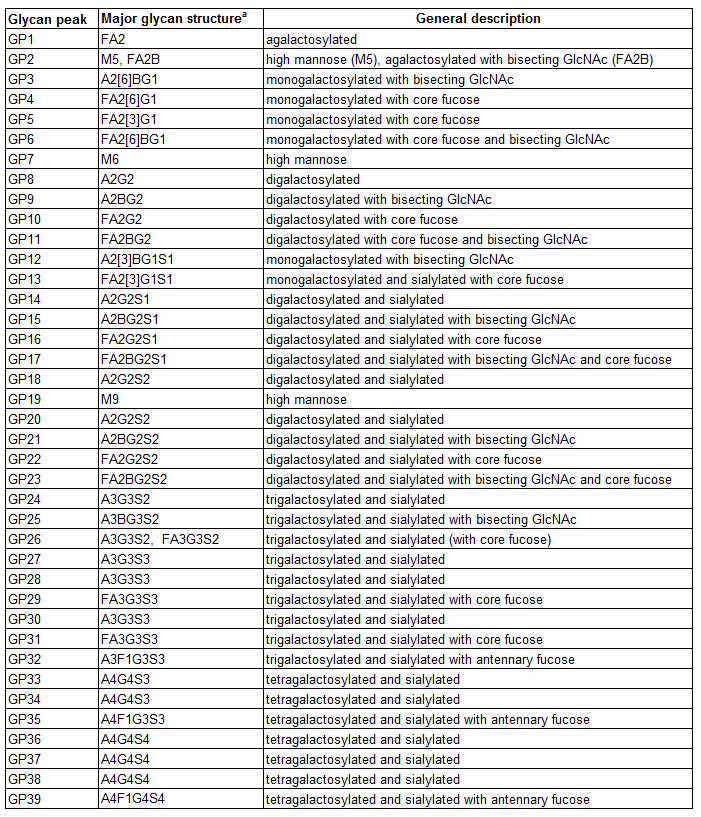


**Supplementary Table 3**. List of major N-glycan structures present in each chromatography peak of plasma N-glycome separated by HILIC-UPLC.

^a^N-glycan structure abbreviations: all N-glycans have two core GlcNAcs; F at the start of the abbreviation indicates a core fucose alpha 1-6 linked to the inner GlcNAc; Mx, number (x) of mannose on core GlcNAcs; Ax, number of antenna (GlcNAc) on trimannosyl core; A2, biantennary with both GlcNAcs as 1-2 linked; A3, triantennary with a GlcNAc linked 1-2 to both mannose and the third GlcNAc linked 1-4 to the alpha 1-3 linked mannose; A4, GlcNAcs linked as A3 with additional GlcNAc 1-6 linked to alpha 1-6 mannose; B, bisecting GlcNAc linked 1-4 to 1-3 mannose; Gx, number (x)of 1-4 linked galactose on antenna; F(x), number (x) of fucose linked alpha 1-3 to antenna GlcNAc; Sx, number (x) of sialic acids linked to galactose ^1^.


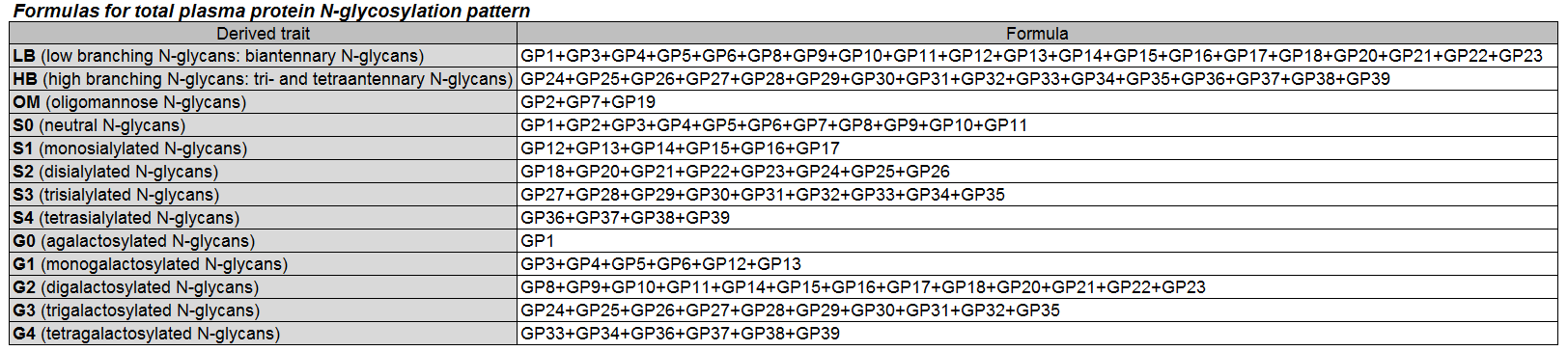

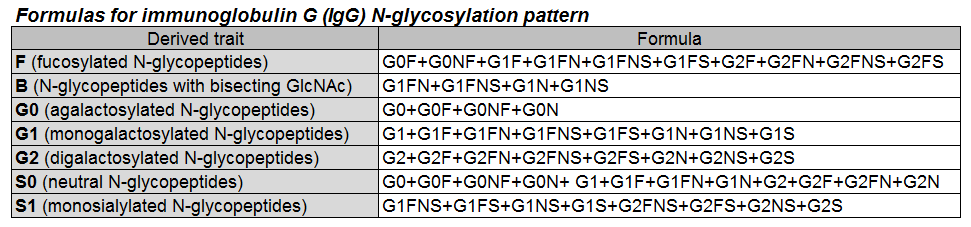


**Supplementary Table 4**. Formulas for N-glycosylation patterns.

**Reference**
